# Visual simulations of presbyopic corrections through cataract opacification

**DOI:** 10.1097/j.jcrs.0000000000001040

**Published:** 2022-08-12

**Authors:** Xoana Barcala, Amal Zaytouny, Daniela Rego-Lorca, Julia Sanchez-Quiros, Ruben Sanchez-Jean, Jose Maria Martinez-de-la-Casa, Carlos Dorronsoro, Susana Marcos

**Affiliations:** From the Institute of Optics, Spanish National Research Council, IO-CSIC, Madrid, Spain (Barcala, Zaytouny, Dorronsoro, Marcos); 2EyesVision SL, Madrid, Spain (Barcala, Dorronsoro); Servicio de Oftalmología, Hospital Clinico San Carlos, Universidad Complutense de Madrid, Spain (Rego-Lorca, Sanchez-Quiros, Sanchez-Jean, Martinez-de-la-Casa); Center for Visual Science, The Institute of Optics, Flaum Eye Institute, University of Rochester, Rochester, New York (Marcos).

## Abstract

To study the viability of visual simulation of presbyopic correction in patients with cataract and the effect and impact of the cataract on the perceived visual quality of the different simulated presbyopic corrections preoperatively and postoperatively.

**Setting:**

San Carlos Clinical Hospital, Madrid, Spain.

**Design:**

Observational, noninterventional, pilot study, early feasibility of the device being studied.

**Methods:**

Cataract patients were tested preoperatively (n = 24) and postoperatively (n = 15) after bilateral implantation of monofocal intraocular lenses (IOLs). The degree of cataract was evaluated objectively with the objective scatter index (OSI). Visual acuity (VA) and perceived visual quality of natural scene images (Multifocal Acceptance Score) were measured before and after cataract surgery at far (4 m), intermediate (64 cm) and near distance (40 cm) with 4 binocular presbyopic corrections (single vision, bifocal, monovision and modified-monovision) simulated with a binocular Simultaneous Vision simulator based on temporal multiplexing.

**Results:**

VA was significantly correlated with OSI (*r* = −0.71, *P* < .0005), although the visual degradation at far for each correction was constant and not correlated with OSI. The visual benefit at near distance provided by the presbyopic correction was noticeable (23.3% ± 27.6% across corrections) for OSI <5. The individual perceptual scores were highly correlated preoperatively vs postoperatively (*r* = 0.64, *P* < .0005) for all corrections and distances.

**Conclusions:**

Visual simulations of IOLs are an excellent tool to explore prospective postoperative vision. The high correlation in the perceptual scores pre- and post-cataract surgery demonstrates that SimVis Gekko can be used in cataractous patients to guide the selection of the optimal correction for a patient.

## Abstract 2

Patients can rank the perceived image quality through cataract up to high level of opacity and that the preoperative ranking of the corrections matches that performed postoperatively through transparent media.

Visual simulators allow noninvasive manipulation of the optics of the eye, providing patients with the visual experience of different optical corrections (eg, corrected aberrations, the optics of another patient, or a range of ophthalmic corrections).^1–4^ The presentation of presbyopic corrections is a prime application of visual simulators. Visual simulators represent a new visual experience for the patient that is generally difficult to imagine. They allow dynamic comparisons of different corrections, helping doctors to select the correction with which the patient obtains optimal vision.^5–7^

Traditionally, visual simulators are based on adaptive optics (AOs), using a deformable mirror and/or a spatial light modulator (SLM) to spatially mimic a given correction. In earlier work using a custom-developed AO system, we have proved the accuracy of an SLM to replicate commercial multifocal intraocular lenses (mIOLs) and contact lenses (CLs).^1,7–9^ To our knowledge, there is at least 1 commercial visual simulator (VAO by Voptica SL) which uses SLM to replicate monocular ophthalmic corrections.^10^

Simultaneous vision simulators are an alternative to AO simulators, which specifically target the simulation of multifocal lenses by superposition of images at different foci.^11,12^ Recent deployments of these systems (Sim+Vis technology) are based on temporal multiplexing using tunable lenses (TLs).^7,13–15^ In these systems, the eye is exposed to a periodic variation (in time) of the optical power without a change in the magnification of the retinal image. By sweeping the power at a frequency higher than the eye's fusion frequency (50 Hz), it is possible to produce a static image on the retina, formed by the superposition of images with different defocus.^16^ By modifying the temporal pattern driving the TL, it is possible to tailor the through-focus performance of multifocal (diffractive and refractive) and extended depth of focus IOLs.^14^ The small footprint of the TL-based temporal multiplexing Sim+Vis technology has allowed building binocular wearable devices (SimVis Gekko, 2EyesVision SL) that are able to simulate various presbyopic binocular corrections, including monovision or modified monovision.^7,9,17,18^

Typically, preoperative simulations of postoperative vision with IOLs are administered on phakic patients with clear lens. Artal et al. showed that the aberrations of the crystalline lens played a secondary role in AO IOL visual simulations.^19,20^ Our previous work using SLM-based and SimVis-based simulations in a bench-top system, as well as a binocular SimVis Gekko, showed excellent correspondence in through-focus vision with simulated trifocal IOLs in preoperative patients with clear lens and the same patients postoperatively with implanted trifocal IOLs (root mean square visual acuity [VA] difference 0.06 ± 0.01).^18^ A subsequent study showed similarly shaped through-focus VA curves with SimVis-simulated IOLs in patients with cataract and with the implanted IOL postoperatively (0.87 shape similarity curve metric) but shifted by a mean of 0.3 decimal VA (absolute value) because of the effect of the scattering caused by the cataract. These results suggested that the SimVis simulations capture the relative through-focus performance of the multifocal lenses and that the absolute postoperative performance could be projected from the preoperative measurements by a single factor accounting for the VA improvement resulting from the elimination of opacity. The temporal multiplexing principle in SimVis allows projecting on the retina the multifocal profile avoiding selective spatial occlusions that will occur in a spatial representation of the lens in other simulator modalities (eg, using an SLM) in patients with cataract.

Even if refractive clear lens exchange procedures are increasingly popular, the implantation of IOLs is still primarily performed on patients with cataract.^21,22^ With the growing choice of IOLs at the patient's disposal, the cataract population would be largely benefited from experiencing vision and judging visual quality with different IOLs, which is now possible with visual simulators. Even if the visual quality is reduced by the presence of the cataract, it would be valuable for the patients to be able to provide a relative assessment of the visual quality of different lenses at various distances, provided that the established relative ranking is accurate.

In this study, we tested the applicability of visual simulations of presbyopic corrections in patients with cataract with different levels of scattering. Patients performed the same tests through simulations of a series of corrections preoperatively (through cataract opacification) and then postoperatively through the same simulated corrections and an implanted mIOL. The visual metrics obtained with SimVis Gekko on patients preoperatively were tested as a function of the cataract level as indicated by the objective scatter index (OSI). We evaluated the effect of the presence (and removal) of the cataract on the visual scoring of images and the ranking of different presbyopic corrections.

## METHODS

Patients (before and after cataract surgery) judged the visual quality of real scene images with the Multifocal Acceptance Score to Evaluate Vision (MAS-2EV) metric at far (4 m), intermediate (64 cm), and near (40 cm) distances with 4 binocular presbyopic corrections simulated with SimVis Gekko: single vision (monofocal far), bifocal, monovision, and modified monovision (dominant eye with monofocal far and nondominant eye with a bifocal lens).

### Patients

Thirty patients scheduled for bilateral cataract surgery with monofocal-far IOLs at the Hospital Clinico Universitario San Carlos in Madrid were recruited for this study. Inclusion criteria included age ranging between 50 years and 85 years, distance spherical equivalent from subjective refraction in the range of +4.00 to −6.00 diopters (D) in each eye, and cylinder ≤2.50 D. Six patients did not meet the inclusion criteria because of high myopia and astigmatism and were discarded from this study. Figure 1 summarizes the demographic and refractive profiles of the 24 patients preoperatively (mean age: 70 ± 10 years, Rx: −0.66 ± 2.21 D, cyl: −1.24 ± 0.65 D). Patients in cells shaded in gray were also measured postoperatively.

Figure 1.Profile of the participating cataract patients. Columns stand for the following: patient ID, sex (F and M), age (years), spherocylindrical refraction (sphere [sph] and cylinder [cyl] in D, and astigmatic angle in degrees) for the right (OD) and left (OS) eyes, monocular decimal CDVA, eye preference, OSI for each eye and mean OSI, and severity identified by asterisks (*: small cataract; **: mild cataract; ***: severe cataract).

**Figure:**
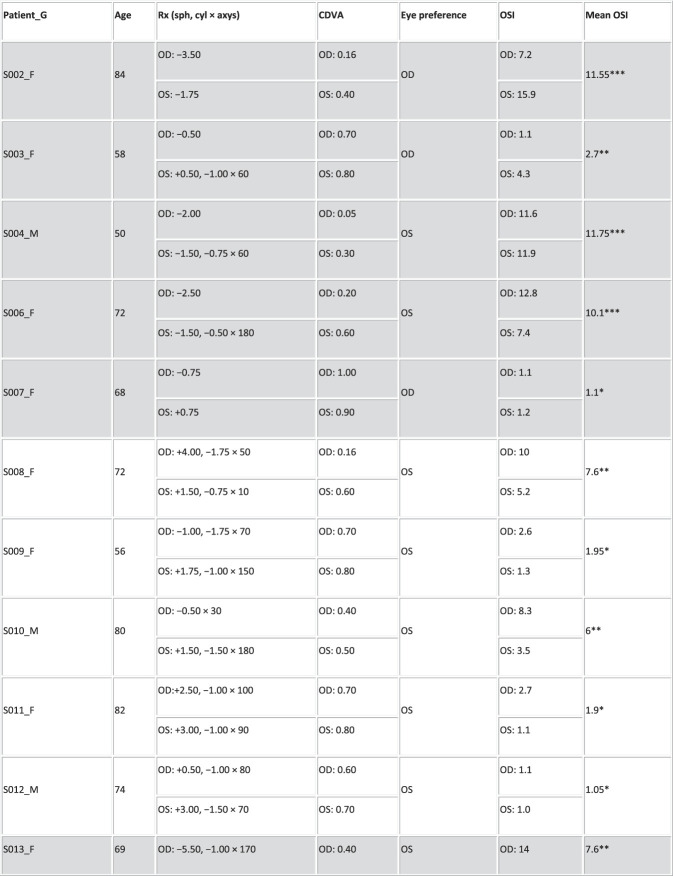

**Figure:**
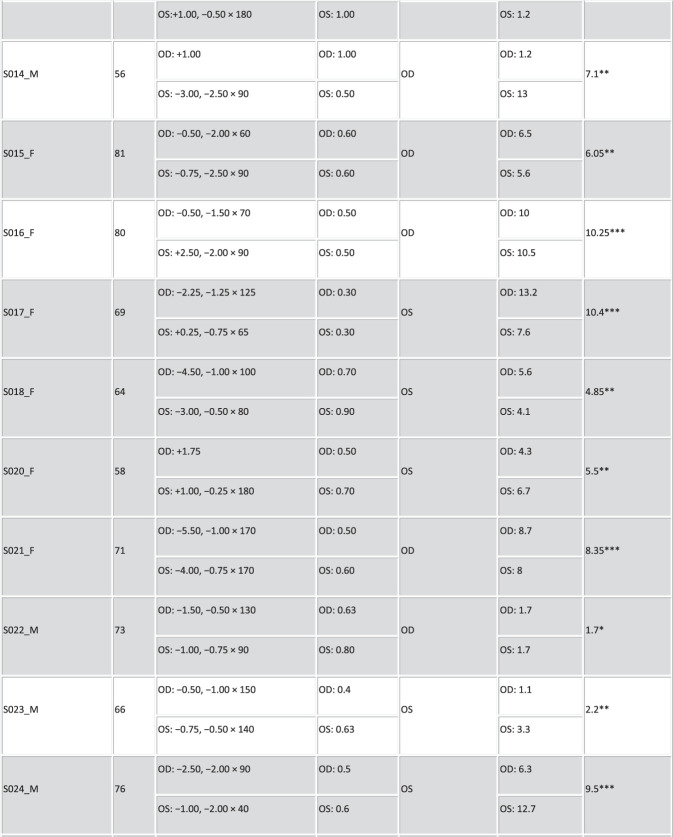

**Figure:**
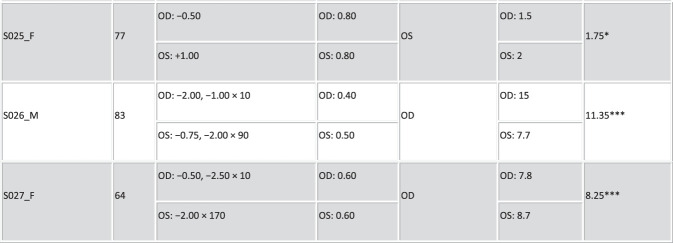

### Cataract Surgeries

Fifteen patients underwent bilateral cataract surgery implanted with mIOLs targeting distance vision correction and were performed under topical anesthesia. IOLs were implanted through a 1.80 mm valving and self-sealing clear corneal incision at 130 degrees at approximately 1 mm anterior to the limbus. Three different types of mIOLs were implanted: Akreos Adapt (Akr., Bausch & Lomb, Inc.; n = 21 eyes), Zeiss CT Asphina (Asp., Carl Zeiss Meditec AG; n = 8 eyes), and TECNIS Monofocal 1-piece aspheric IOL (Tec., Johnson & Johnson Vision; n = 1 eye). The IOL power ranged from 13 to 23 D. The selected IOL was then implanted in the capsular bag with a single-use injection system, Viscoject 1.8 from Bausch & Lomb, Inc. (used for the Akreos Adapt IOL) and BLUEMIXS 180 from Zeiss (used for the Zeiss CT Asphina IOL). Optical biometry was conducted using IOLMaster 700 (Carl Zeiss Meditec AG). The Barrett TK Universal II formula was used to select IOL power, with A-constants 118.5, 118.0, and 119.3 for the Akreos, Asphina, and TECNIS IOLs, respectively.

The patients underwent standard postoperative clinical evaluations 1 day, 1 week, and 1 month after unilateral surgery. Surgeries were performed 1.7 ± 1.25 months apart between the first and second eyes, except for 4 patients (S003, S006, S021, and S025) who had 14.5 ± 2.65 months between surgeries. Postoperative visual simulations were performed 3 to 8 months after surgery of the second eye, on average. A slitlamp evaluation was performed at the time of postoperative measurement to ensure that no posterior capsular opacification had been developed or to discard other potential complications/artifacts in the IOL such as whitening or calcification (which did not occur in any patient).

Figure 2 presents the profile of the patients (mean age: 70.33 ± 9.24 years, implanted mIOL and mean residual subjective refractive error; sph: −0.37 ± 0.73 D and cyl: −0.43 ± 0.52 D). All patients achieved 0.90 or better decimal corrected distance VA for far distance, except for S006.

Figure 2.Profile of the postoperative patients. Columns stand for the following: patient ID, sex (F and M), implanted monofocal IOL (Akr. stands for Akreos Adapt, Asp. for CT Asphina, and Tec. for TECNIS Monofocal), age (years), spherocylindrical residual refraction (sphere [sph] and cylinder [cyl] in D, × astigmatic angle in degrees) for the right (OD) and left (OS) eyes, monocular decimal VA, and eye preference (surgery flipped ocular preference highlighted in blue).

**Figure:**
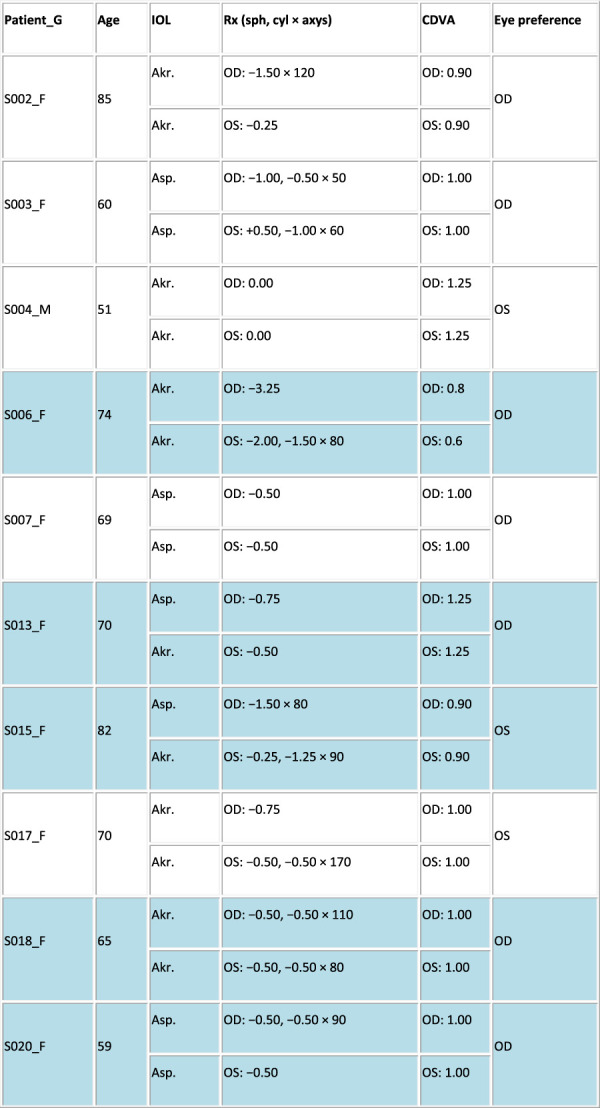

**Figure:**
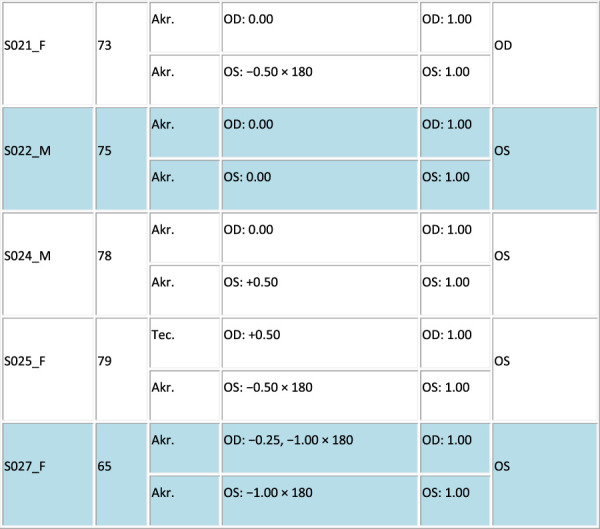

### Preoperative Cataract Scattering

The OSI obtained using the HD Analyzer (Visiometric SL) was used to quantify the degree of opacification in cataracts. This instrument is based on the double-pass technique that provides an objective clinical evaluation of the optical quality of the eye.^23–25^ In this double-pass technique, the light (near-infrared light, 780 nm) coming from a point of source forms the image on the retina, and it is reflected and passed through the eye's optics twice using an unequal pupil (small pupil in the way in and a large pupil in the way out). The OSI is calculated from the aerial retinal image as the ratio of the intensity at an eccentric location (between 0.2 degrees and 0.33 degrees) and the central part.^26^ The higher the OSI value, the greater the level of intraocular scattering.

According to the OSI manual user (v. 2.7-2018), an OSI value of ≤1 is indicative of normal scattering, OSI between 1.5 and 4 for eyes with developing cataracts, and OSI >4 for mature cataracts. Based on the measurements presented above, patients were classified into 3 different groups based on the mean OSI: small cataracts with OSI < 2, mild cataracts when OSI is between 2 and 8, and mature cataracts when OSI is above 8.

The OSI measurements were obtained before cataract surgery of the first eye in the same session as the visual simulations. The preoperative OSI values in all patients are summarized in Figure 1.

### Sensory Ocular Dominance (Preferred Eye) and Binocular Vision Test

Sensory ocular dominance was determined by placing a +1.50 D lens alternatively in 1 eye, which was repeated 3 times.^27^ The patients are asked to look at lines above their best subjective far VA. The preferred eye is identified as the one with which the patient experiences a higher uncomfortably blurred visual percept on introduction of the defocusing lens. The preoperative and postoperative ocular preferences in all patients are summarized in Figures 1 and 2, respectively. Natural binocular vision was tested using a 4-dot Worth test to rule out fusion dysfunction.

### SimVis Gekko

SimVis Gekko (2EyesVision) is a binocular wearable visual simulator (Supplemental Figure 1A, http://links.lww.com/JRS/A673) based on the principle of temporal multiplexing.^13,15^ The system is see-through (Supplemental Figure 1B, http://links.lww.com/JRS/A673) and exhibits a 20-degree field of vision in each eye. The unit is operated wirelessly from an iPad, which sends the corrections and guides the measurement.

### Simulated Presbyopic Corrections

Four binocular presbyopic corrections were tested using SimVis Gekko v.1.5: (1) single vision or monofocal far in both eyes (FF), (2) bifocal lenses in both eyes (BB; Supplemental Figure 1D, http://links.lww.com/JRS/A673), (3) monovision (FN; preferred eye with monofocal far and nonpreferred eye with monofocal near), and (4) modified monovision (FB; preferred eye with monofocal far and nonpreferred eye with a bifocal lens). The bifocal correction represented a generic bifocal IOL with +2.50 D near add. The generic bifocal correction simulates a 50/50 energy split between far and near visions. In this correction, the TL spends half of the time in an optical power corresponding to far vision and half of the time in an optical power corresponding to near vision (with 2.50 additional diopters) in cycles of 50 Hz. The temporal coefficients driving the lens also correct for the dynamic effects of the TL, accounted by its impulse response measured with a high-speed focimeter.^28^ The resultant through-focus optical curve of the bifocal simulated correction exhibits 2 narrow peaks of the Strehl ratio centered at 0.00 D and 2.50 D. In the monovision and modified monovision, the near add imposed in the nonpreferred eye was 2.50 D while the dominant eye was given a monofocal correction focused at far.

### Modified MAS-2EV

The Multifocal Acceptance Score to Evaluate Vision (MAS-2EV) is a recently published metric to evaluate the global quality of vision, and it is particularly well suited to assess perceived visual quality with presbyopic correction in patients with clear lens.^29^ The metric comprises 5 perceptual scores (PSs) of multistimuli images of day and night scenes, at far (4 m) and near (40 cm) distances, and a stereovision target at near. The patient gives PSs according to his/her perceived image quality of the visual scenes through a correction from very blurred (PS: 0) to very sharp (PS: 10).^30^

For this study, the MAS-2EV was modified to evaluate only the images for daylight conditions and by introducing an intermediate image at 64 cm of distance. Supplemental Figure 2 (http://links.lww.com/JRS/A674) shows MAS-2EV far, intermediate, and near images used in this study. The images subtend a 15.24-degree field, a 20.55-degree field, and a 30.96-degree field horizontally for far, intermediate, and near, respectively. Far images were displayed on a 43″ monitor (LG) and the intermediate and near ones on an iPad Pro of 12.9″ with Retina Display (Apple, Inc.). Measurements were performed under photopic conditions (≈85 cd/m^2^).

Patients judged the perceptual quality of images at far, intermediate, and near (PSs: 0 to 10) through the SimVis Gekko simulating the IOL designs: monofocal far (both eyes; FF), bifocal (both eyes; BB), monovision (far in the dominant eye and near in the contralateral eye; FN), and modified monovision (far dominant eye/bifocal contralateral eye; FB).^30^ Three repetitions were performed for each correction at each distance.

Near stereoacuity targets are presented, consisting of a random-dot anaglyph with different rectangles in different positions and with different crossed disparities (400 to 40 seconds of arc [arcsec]). The anaglyphs are presented in an iPad Pro of 12.9″ with Retina Display (by Apple, Inc.) and observed with cyan/red spectacles. Near stereoacuity was measured a single time for each correction at 40 cm. The measured stereopsis was converted into a 0 to 10 scale, 40 arcsec being equivalent to a score of 10 and 400 arcsec equal to a score of 0.

### Experimental Protocol

This study was conducted at Hospital Clinico San Carlos, and the measurements were conducted by 2 experienced optometrists (X.B. and A.Z.). Measurements were performed under natural viewing conditions (natural pupil size).

An experimental session included the following sequence: (1) subjective refraction, (2) eye preference, (3) binocular vision, (4) OSI measurement, and (5) MAS-2EV with SimVis Gekko. The same sequence of tests was followed preoperatively and then repeated 3 to 8 months after the surgery of the second eye.

A preparatory trial using SimVis Gekko allowed the patient to set the range for their perceptual scoring by viewing the MAS-2EV far-daylight images through a simulated far-distance corrected monofocal lens (10 PS) and an additional 2.50 D monofocal lens (0 PS). The patient's spherocylindrical refraction was corrected by using trial lenses placed in the dedicated slot in the SimVis Gekko.

**Figure 3.**
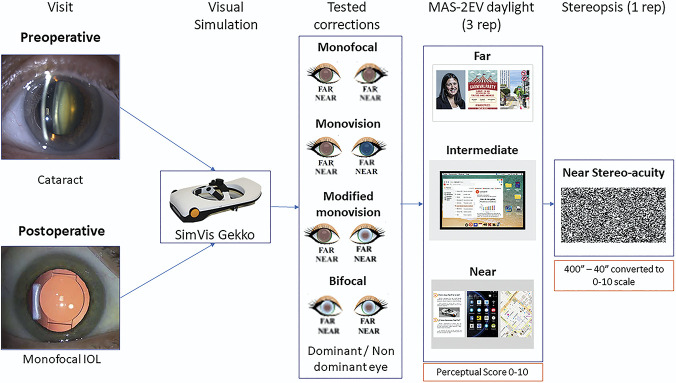
Methods overview. Patients, before and after cataract surgery (with implantation of a mIOL), were measured with 4 corrections simulated through SimVis Gekko: monofocal (corrected for far in both eyes, FF), monovision (dominant eye with monofocal far and nondominant eye with monofocal near of +2.50 D, FN), modified monovision (dominant eye with monofocal-far and nondominant eye with a bifocal lens of +2.50 D of addition, FB), and bifocal (corrected with generic bifocal lenses of +2.50 D addition in both eyes). Patients judged the visual quality of MAS-2EV images at far, intermediate, and near (daylight) and performed a near stereopsis test.

Figure 3 shows the methodology followed in this study. The 4 binocular presbyopic corrections were tested using SimVis Gekko in a random order. For a given correction, stereopsis at near was first tested (yielding a 0 to 10 score). Then, modified MAS-2EV scorings were obtained for far daylight and near daylight images. Before any measurement, patients signed an informed consent form after receiving an explanation of the nature and implications of this study. The approval from the Institutional Review Board, Hospital Clinico San Carlos Ethics Committee, was obtained. The experiments conformed to the tenets of the Declaration of Helsinki.

### Data Analysis

The following metrics were calculated from the MAS-2EV with all tested corrections: visual degradation at far defined as follows: (PS Far for each presbyopic correction − PS Far FF)/PS Far FF, and visual benefit at near defined as follows: (PS Near for each presbyopic correction − PS Near FF)/PS Far FF.^29^ Estimates of the visual degradation of the VA at far and the visual benefit at near were correlated with OSI to evaluate dependencies of the effect of cataract on the performance of the correction (linear regression, Pearson coefficient of correlation *r*, and *P* value).

MAS-2EV PS was analyzed for each distance separately and correlated with OSI. MAS-2EV PSs were compared preoperatively and postoperatively to evaluate to what extent the preoperative assessment of different presbyopic corrections (through the cataract) matches the postoperative assessment of the same corrections (through a clear lens).

## RESULTS

### SimVis Gekko Simulations in Cataract Patients

The cataract patients spanned a wide range of scattering levels (OSI from 1.05 to 11.75, Figure 1). There were also differences in OSI between eyes of the same patient. It was found that VA was significantly correlated with OSI (*r* = −0.71, *P* < .0005). In addition, the OSI difference between eyes was significantly correlated with the VA difference between eyes (*r* = −0.60, *P* = .002).

**Figure 4.**
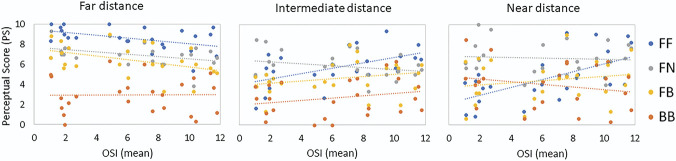
Perceptual score as a function of the mean OSI (mean between right and left eyes) at far (*left*), intermediate (*middle*), and near (*right*) distances for monofocal-far (FF) correction (*blue*), monovision (FN, *gray*), modified monovision (FB, *yellow*), and bifocal in both eyes (BB, *orange*). OSI = objective scatter index

Figure 4 shows the dependence of the PSs on the OSI for far, intermediate, and near distances. Since the PSs were obtained binocularly, the mean OSI value (between right and left eyes) was used. As expected, at far distance, the PSs decreased significantly with increasing OSI for most corrections (FF: *r* = −0.5, *P* = .02; FN: *r* = −0.4, *P* = .05; FB: *r* = −0.4, *P* = .04). Conversely, the PSs at near and intermediate are positively correlated with increasing OSI with the monofocal-far correction (*r* = 0.5, *P* = .007 for intermediate distance and *r* = 0.6, *P* = .002 for near distance).

**Figure 5.**
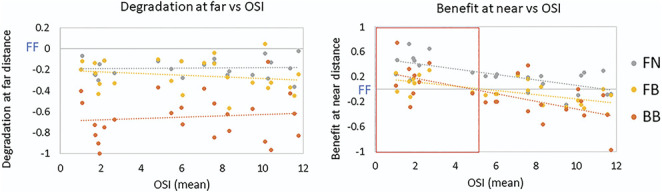
Visual degradation at far distance (left graph) and visual benefit at near distance (right graph) as a function of the (interocular) mean OSI. OSI = objective scatter index

Figure 5 shows the OSI dependence of the visual degradation at far distance for all presbyopic corrections. There was a constant visual degradation at far for a given correction, regardless of OSI. For OSI <5, there was a mean visual degradation of −36.4% ± 27.2% and, for OSI >5, a mean visual degradation of −36.1% ± 25.6%. The visual benefit at near distance provided by the presbyopic correction was apparent (23.3% ± 27.6% on average across corrections) for OSI <5. On average, an OSI of 5 was found to correspond with a decimal VA of 0.50.

### Preoperative and Postoperative Perceptual Scores

Fifteen patients were measured both preoperatively and postoperatively. The OSI in this subset of patients ranged from 1.1 to 11.75 preoperatively (Figure 1, shaded cells). Surgery flipped ocular preference in 46.67% of patients (Figure 2, highlighted in blue). It was found that 57.14% of the patients with flipped ocular preference had higher interocular OSI differences (range 2.4 to 12.8, mean of 4.33 ± 6.49), and 42.86% of the patients had an interocular OSI difference ≤1.00.

On average across distances, corrections, and patients, scores increased by 0.66 points from the preoperative to postoperative measurements. The increase in score was statistically correlated with OSI since, as expected, the patients with the highest OSI scores provided the lowest scores preoperatively.

Supplemental Figure 3 (http://links.lww.com/JRS/A675) shows the monofocal and presbyopic corrections, ranked from highest to lowest modified MAS-2EV scores, preoperatively (through cataract opacification) and postoperatively (through the implanted IOL). Corrections are ranked according to the preoperative PS (averaged across patients) at far. Patients ranked the perceived quality with these corrections in the same order preoperatively and postoperatively for each distance, although there was a systematic increase in the scores after cataract surgery (2.48% for FF, 9.80% for FN, 9.96% for FB, and 3.90% for BB).

**Figure 6.**
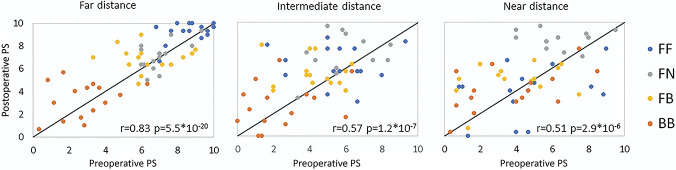
Correlation between the PSs preoperatively and post-operatively across patients and corrections for far, intermediate, and near distances. PS = perceptual score

Figure 6 shows the pooling of all individual PSs for all corrections and distances preoperatively vs postoperatively. The high statistical correlation (*r* = 0.64, *P* < .0005) indicates that patients are accurate at ranking the perceptual quality of the images with different corrections even through cataract opacification. We repeated the same analysis at all distances, comparing the scores across corrections at a given distance preoperatively and postoperatively. At far, we found a high correlation between preoperative and postoperative scores (*r* = 0.83, *P* < .0005). The correlation between preoperative and postoperative PSs is slightly reduced at intermediate (*r* = 0.57, *P* < .0005) but still highly significant. However, we did not find a significant correlation between the absolute PSs preoperatively and postoperatively at near distance (*r* = 0.51, *P* < .0005). The slope <1 in these regressions (s = 0.63 at all distances; s = 0.80 at far; s = 0.50 at intermediate; s = 0.50 at near) is indicative of an increase in the absolute magnitude of the scores postoperatively.

## DISCUSSION

This study demonstrates the viability of visual simulators, particularly the SimVis Gekko, in patients with cataract. The results demonstrate that in patients with mild and moderate cataract, the relative visual quality scores given to different presbyopic corrections preoperatively are preserved when the cataract is removed. This indicates that visual simulators can be reliably used to guide the optimal presbyopic correction for a patient, even in the presence of cataracts. A limitation of this study could have been the use of cataract classification using only the OSI from the HD Analyzer. On the one hand, the double-pass (or rather one-and-half) principle of the HD Analyzer uses a small pupil on the way into the eye and a large pupil for light exiting the eye. Therefore, the OSI metric seems to be directly related to the amount of backward scattering, and its direct relation to vision will hold as long as backward and forward scattering are correlated.^26^ The measurement could be affected by the artifact of infrared light diffusion in the choroid, easily penetrated by this wavelength light, which can be considered a relatively constant background.^31^ Some proposed alternatives modified the double-pass technique by adding a green light source (530 nm), obtaining images with uniform radiance and not influenced by the reflection from the deep choroid.^32,33^ Contrarily, the metric accounts for an overall measure of the contrast reduction produced by the opacities of the crystalline lens, regardless of their spatial distribution. Although the analysis of retinal aerial images from techniques that scan the pupil on different locations, such as ray-tracing techniques, could allow a spatially resolved mapping of the opacification across the pupil, that degree of granularity is not needed in the current application.^34^ The SimVis Gekko works under a temporal multiplexing principle and does not use a spatial mapping of the lens. Therefore, the image rendered on the retina, although degraded by scattering, is not affected by specific spatial coupling of the lens profile and opacity distribution. A previous study showed a great correlation between the iTrace Dysfunctional Lens Index and the OSI.^35^

An alternative metric to grade cataracts is with the Lens Opacities Classification System III (LOCSIII).^36^ The LOCSIII is a subjective scale provided by the operator from analysis of the color and opalescence of slitlamp photographs obtained under dilated pupils through the slitlamp. In LOCSIII, the lens opacifications are classified into 3 different groups: nuclear, cortical, and posterior subcapsular. Previous studies have demonstrated statistical correlation between the LOCSIII scale and the OSI.^33,37,38^ In particular, Lago et al. analyzed 85 cataractous eyes using both the LOCSIII scale and the OSI.^39^ Both scales were statistically correlated for nuclear (*P* = .001), cortical (*P* = .004), and posterior subcapsular (*P* = .001) opacities.

Another limitation of this study might have been the relatively large difference in opacification between the 2 eyes of the same patient (ie, S002, S006, S013, S014, S017, S024, and S026). Since SimVis Gekko and MAS-2EV are naturally binocular measurements, we chose to calculate mean OSI between the left and right eyes to account for an overall reduction by the cataract and to analyze the dependence of the perceptual scores with each correction on the OSI. Alternative analysis may have involved assuming that the perceived image quality is driven by the eye with the least degradation. In fact, previous work has found that binocular adaptation of blur is biased by the sharper of the 2 eyes' retinal images in patients with interocular differences of optical degradation.^40^ When the maximum OSI was correlated with the PSs at far distance, we could not find a statistically significant decrease in PSs with increasing maximum OSI for any of the corrections (*P* > .10). Conversely, at far distance, the PSs decreased significantly with increasing mean and minimum OSI for most corrections. However, the PSs at near distance positively correlated with increasing OSI (maximum, minimum, and mean) with the monofocal-far correction (*P* = .016, *r* = 0.61 for minimum OSI; *P* = .01, *r* = 0.51 for maximum OSI and *P* = .0017, *r* = 0.6 for mean OSI). The analysis is complicated by the fact that 2 of the 4 tested corrections involve monovision. Monovision corrections are applied such that the dominant eye is corrected for far and the nondominant eye is corrected for near (and the bifocal correction, in modified monovision). In previous work on patients with clear lens, we found significant differences in MAS-2EV with monovision, simply flipping the eye in which the corrections are applied.^41^ The shift in measured ocular preference postoperatively suggests that the difference in opacity between the left and right eyes may be limiting the identification of the dominant eye. In a previous study by Schwartz et al., the ocular dominance was measured with the sighting dominance test before and after unilateral cataract surgery.^42^ They did not grade the cataract with any other metric but VA. Only 21% of the patients had a change in ocular dominance, which was exclusively correlated with the best VA eye (in 80% of the patients). We found a statistical correlation between the OSI interocular difference and the difference in PSs before and after cataract surgery and/or ranking. The potentially temporary shift in ocular preference because of the presence of cataract is likely a contributor to the reduced performance of a given correction. Apart from OSI interocular differences and a large OSI range in our samples, other differences between sessions and across patients may arise from differences in the period of time between eye surgeries (1 to 3 months in 73.3% of the patients, and 11 to 18 months in 26.7% of the patients) or susceptibility of the selected ocular preference method to interocular differences of blur. Other sensory dominance methods, have recently reported that use of the SimVis Gekko itself may be more accurate than routine ocular dominance tests used in the clinic.^43^ An interesting finding of our study is the ability of patients with cataract to rank the different corrections through cataract opacification (OSI from 1.05 to 11.75, *P* > .005). Patients can also judge the visual benefit at intermediate and near with the presbyopic corrections with OSI up to 5 (0.5 decimal VA).

An unexpected result of this study is the fact that relatively high scores were given by patients with cataract at intermediate and near distances with a monofocal-far correction. We found that the PS at intermediate and near with the monofocal correction at far increases with the OSI. This effect may be associated with an increase in blur tolerance in patients with a higher amount of scattering. The origin can be optical, as suggested by Artal et al., in a study showing beneficial optical interactions between aberrations and scattering.^44^ Contrarily, increased perceived focus of blurred images is found in patients following neural adaptation to blur.^45^ Although, to our knowledge, most of the previous literature refers to adapting images blurred by Gaussian blur, pure defocus, or higher-order aberrations, it would not be surprising that similar mechanisms also apply to blur produced by cataract, which (as the other forms of blur) leads to a loss in contrast.

In summary, visual simulations of IOLs are an excellent tool to explore prospective postoperative vision. The high correlation in the perceptual scores before and after cataract surgery demonstrates that the SimVis Gekko can be used in cataractous patients.

**WHAT WAS KNOWN**

- Visual simulators allow dynamic comparisons of different corrections, such as presbyopic corrections.
- The implantation of IOLs is still primarily performed on patients with cataract, in which the visual quality is reduced by the presence of the cataract.
- To our knowledge, most visual simulators do not provide an accurate IOL simulation because of their working principle and the presence of the cataract, depriving cataract patients to assess their visual quality with different presbyopic corrections.

**WHAT THIS PAPER ADDS**

- SimVis Gekko, a binocular see-through visual simulator, allows patients with cataract (with different levels of scattering) to fully experience presbyopic corrections.
- Patients experience and rank different presbyopic corrections preoperatively (with the presence of the cataract) and postoperatively (implanted with a mIOL). The high correlation in the perceptual scores before and after cataract surgery demonstrates that SimVis Gekko can be used in cataractous patients to guide the selection of the optimal correction for a patient.
